# Angiotensin-Converting Enzyme Inhibition and/or Angiotensin Receptor Blockade Modulate Cytokine Profiles and Improve Clinical Outcomes in Experimental COVID-19 Infection

**DOI:** 10.3390/ijms26167663

**Published:** 2025-08-08

**Authors:** Yasmin da Silva-Santos, Roberta Liberato Pagni, Thais Helena Martins Gamon, Marcela Santiago Pacheco de Azevedo, Maria Laura Goussain Darido, Danielle Bruna Leal de Oliveira, Edson Luiz Durigon, Maria Cecília Rui Luvizotto, Hans Christian Ackerman, Claudio Romero Farias Marinho, Leonardo José de Moura Carvalho, Sabrina Epiphanio

**Affiliations:** 1 Laboratory of Cellular and Molecular Immunopathology of Malaria, Department of Clinical and Toxicological Analysis, Faculty of Pharmaceutical Sciences, University of São Paulo, São Paulo 05508-000, Brazil; yasmins@usp.br (Y.d.S.-S.); sabrinae@usp.br (S.E.); 2 Laboratory of Malaria Research, Oswaldo Cruz Institute, Oswaldo Cruz Foundation, Rio de Janeiro 21040-900, Brazil; 3 Immunology Laboratory, Heart Institute, Faculty of Medicine, University of São Paulo, São Paulo 05403-000, Brazil; ropagni@usp.br; 4 Laboratory of Clinical and Molecular Virology, Institute of Biomedical Sciences, Department of Microbiology, University of São Paulo, São Paulo 05508-000, Brazil; thagamon@usp.br (T.H.M.G.); marcela.spachecoazevedo@gmail.com (M.S.P.d.A.); mlauradarido@icb.usp.br (M.L.G.D.); danibruna.inovatech@gmail.com (D.B.L.d.O.); eldurigo@usp.br (E.L.D.); 5 Laboratory of Experimental Immunoparasitology, Institute of Biomedical Sciences, Department of Parasitology, University of São Paulo, São Paulo 05508-000, Brazil; marinho@usp.br; 6 Hospital Israelita Albert Einstein, São Paulo 05652-900, Brazil; 7 School of Veterinary Medicine of Araçatuba, São Paulo State University, São Paulo 16050-680, Brazil; celuvizotto@gmail.com; 8 Physiology Unit, Laboratory of Malaria and Vector Research, National Institute of Allergy and Infectious Diseases, Rockville, MD 20852, USA; hans.ackerman@nih.gov

**Keywords:** SARS-CoV-2, K18-hACE2 mice, Losartan, lisinopril, COVID-19

## Abstract

The regulation of angiotensin-converting enzyme 2 (ACE2) expression by medications such as ACE inhibitors (ACEis) and angiotensin receptor blockers (ARBs) has raised critical questions regarding their potential benefits and risks during COVID-19. ACE2, a regulator of blood pressure through the renin–angiotensin system (RAS), is the primary receptor for SARS-CoV-2. ACEis and ARBs can modulate ACE2 expression, potentially exacerbating viral load. However, the risks of higher viral load could be mitigated by favorable anti-inflammatory responses associated with ACEi and ARB use, highlighting the complexity of their impact on viral replication and disease outcomes. This study investigates the effects of sustained Losartan monotherapy (ARB) and combination Losartan + Lisinopril (ARB + ACEi) on viral replication, inflammation, lung function, and clinical measures of disease severity in a murine model of severe COVID-19 involving humanized ACE2 transgenic mice infected with SARS-CoV-2 Wuhan strain. Both ARB and ARB + ACEi treatments led to increased ACE2 expression in the lungs and higher viral load post-infection. Despite this, the ARB + ACEi combination improved clinical scores, reduced weight loss and inflammatory cytokine levels, and preserved lung function, though it did not improve survival. Overall, the results of these controlled experiments provide insight into the complex dynamics of ACEi and ARB use in COVID-19; while these drugs induce expression of the ACE2 receptor and increase viral load, they provide compensatory modulation of the inflammatory response that appears to diminish severity of the infection.

## 1. Introduction

The COVID-19 pandemic, caused by SARS-CoV-2 infections that began in December 2019, with the first outbreak detected in Wuhan, a Chinese province, recorded 775 million cumulative cases of the disease worldwide until January 2024 [1], resulting in 7 million recorded deaths [2]. In addition, survivors often suffer from long-term complications in a syndrome referred to as long COVID-19 [3]. The SARS-CoV-2 virus’s high affinity for human cells has contributed to high viral replication and global spread. The interaction between virus and cell begins when the virus spike protein specifically binds to the host cell through the angiotensin-converting enzyme 2 (ACE2) receptor. This binding triggers the fusion of the viral and cellular membranes, allowing the virus to enter and replicate. However, it is essential to emphasize that this same enzyme plays a crucial role in human physiology, especially in regulating the renin–angiotensin system, essential for maintaining blood pressure homeostasis [4,5,6]. This regulation occurs through classical and alternative pathways, balancing the activation of receptors involved in vasoconstriction and vasodilation of blood vessels and other factors with anti-/pro-inflammatory and anti-/pro-fibrotic properties [7,8,9].

Juxtaglomerular cells in the kidneys are responsible for the production of renin, which catalyzes the conversion of angiotensinogen (ANG), produced in the liver, into angiotensin 1 (ANG1). In the classical pathway, ANG1 is converted into angiotensin 2 (ANG2) by the angiotensin-converting enzyme 1 (ACE1). ANG2 activates the angiotensin 1 receptor (AT1R), triggering vasoconstriction and pro-inflammatory/thrombotic activities. On the other hand, in the alternative pathway, the degradation of ANG1 by ACE2 results in the formation of angiotensin 1–9 (ANG1-9) and, subsequently, angiotensin 1-7 (ANG1-7). These ANG1 byproducts exert opposite effects on AT1R activation; when they bind to the MAS receptor, they promote vasodilation and possess anti-inflammatory/anti-thrombotic properties [10,11].

Evidence indicates that angiotensin-converting enzyme inhibitors (ACEis) and/or angiotensin receptor blockers (ARBs) may increase the expression of ACE2 in specific tissues, especially in the lungs [6,12]. The use of these drugs is not restricted to hypertensive patients but is also relevant to patients with cardiac, renal, and diabetic health problems. Treatment with ACEis interferes with the conversion of ANG1 to ANG2 by inhibiting ACE1, reducing AT1R activity, and promoting the formation of ANG1-9. ARBs act by directly blocking the AT1R receptor and furthering the conversion of ANG1 and ANG2 into products of the alternative pathway. Both classes of drugs favor the activation of the MAS receptor. When used together, ACEi + ARB promotes a dual blockade of the classical pathway, suppressing the activation of the receptor with pro-inflammatory/thrombotic activity and promoting the activation of the MAS receptor [13,14].

Individuals who regularly use these medications are also part of the risk group for COVID-19 [15]. During the pandemic, there were uncertainties regarding the continuation or discontinuation of these medications that could modulate the expression of ACE2 potential impact viral entry and replication, as well as impact the balance of the renin–angiotensin system (RAS) [16].

Continuing treatment with ACEis and/or ARBs may lead to increased availability of ACE2, which may favor viral replication. However, discontinuing these medications may exacerbate the patient’s pre-existing condition. Furthermore, the ongoing use of these drugs may be crucial for protecting against more severe forms of the disease, as they promote positive regulation of ACE2, thereby facilitating the ANG1-9 axis [9,16,17]. The use of ACEis and/or ARBs provides vasodilatory and anti-proteinuric effects, offering protection against cardiac complications (such as heart failure, ventricular hypertrophy, and acute myocardial infarction) and renal complications resulting from diabetes mellitus [17,18,19,20,21].

Systematic reviews/meta-analyses have been conducted to clarify the relationship between the use of these medications and COVID-19. The BRACE CORONA trial identified that continued therapy in patients with moderate disease resulted in more days alive and out of the hospital [22]. Similarly, discontinuation of ACEi and/or ARB medications should not be undertaken [23]. Other studies conducted at different medical centers did not find differences in severity/mortality. Moreover, using ACEis and/or ARBs was important for attenuating the inflammatory response, reducing the length of hospital stays, admission to the intensive care unit, and developing severe/long COVID-19 [24,25,26,27,28,29,30].

In a recent study of experimental COVID-19 using SARS-CoV-2 infection in humanized ACE2 transgenic (K18-hACE2) mice, we observed a significant reduction in lung ACE2 levels 3 days post-infection (dpi). Treatment with the ACEi Lisinopril increased baseline lung ACE2 levels before infection and accelerated the recovery of lung ACE2 by 5 dpi; however, Lisinopril resulted in higher lung viral loads at 3 and 6/7 dpi compared to untreated animals. Lisinopril also promoted a rapid recovery of lung ACE2 levels by 5 dpi and significantly reduced pro-inflammatory cytokines IL-6 and TNF-α in both serum and lungs at 6/7 dpi. These effects were accompanied by marginal improvements in body weight, clinical scores, and survival. No differences in respiratory function or lung histology were observed between treated and untreated infected mice. Therefore, Lisinopril treatment exhibited both deleterious effects, such as increased viral loads, and beneficial effects (anti-inflammatory, anti-constrictor, and anti-coagulant). These opposing effects appeared to balance each other, leading to only marginal overall beneficial effects for Lisinopril-treated animals [31].

The present study aims to further investigate the effects of ACE2 modulation through ACEi and ARB treatments in humanized transgenic mice infected with SARS-CoV-2. By using ARBs alone or in combination with ACEis (Losartan + Lisinopril), we seek to elucidate the role of these medications in regulating ACE2 levels, viral replication, and overall disease outcome in experimental COVID-19.

## 2. Results

### 2.1. Losartan and Losartan + Lisinopril Combination Ameliorate Weight Loss and Clinical Manifestations in SARS-CoV-2-Infected Mice

**Figure 1:**
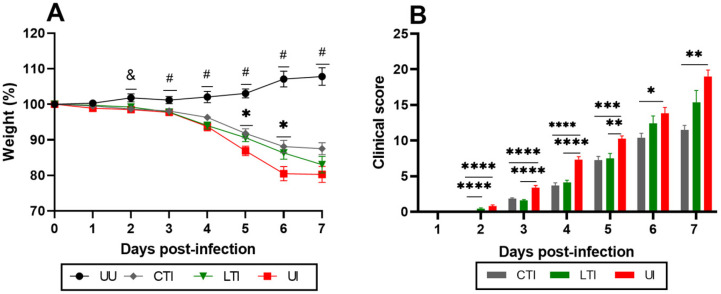
(**A**) Analysis of weight loss (%) and (**B**) clinical score (daily sum) of uninfected control and SARS-CoV-2-infected K18-hACE2 mice treated with Losartan or Losartan + Lisinopril combination. UU = Untreated Uninfected, CTI = Combination-treated Infected, LTI = Losartan-treated Infected, and UI = Untreated Infected. Signs indicate significant differences: (#) all groups versus UU group; (&) UI versus UU group; (*) UI versus CTI or LTI: * *p* < 0.05, ** *p* < 0.005, *** *p* < 0.0005 and **** *p* < 0.0001. Mice in the UU group showed clinical scores of zero at all times. Data presented as mean ± standard error of the mean (SEM). Analysis performed by mixed-effects model with Geisser–Greenhouse correction, followed by Tukey’s post hoc test. *n* = 30 per group.

From 2 dpi, the Untreated Infected group exhibited progressive weight loss compared to healthy mice (p_adj_ = 0.0435) and reached ~25% weight loss at 6 dpi (Figure 1A). While both Losartan-treated and combination-treated infected mice experienced weight loss, the extent of this loss was significantly less pronounced compared to the Untreated Infected group at 5 and 6 dpi (p_adj_ < 0.05).

A similar pattern was observed for clinical manifestations. Untreated Infected mice showed a progressive increase in clinical scores, peaking at ~15 points by 6 dpi. In contrast, the Combination-treated Infected group recorded significantly lower clinical scores throughout the infection (period from 2 to 7 dpi) compared to Untreated Infected mice (*p* < 0.05). In contrast, Losartan-treated Infected mice also demonstrated improved clinical scores from 2 to 5 dpi (*p* < 0.005), though no significant differences were observed at 6 and 7 dpi (Figure 1B). No significant differences in weight loss or clinical scores were observed between sexes across all groups and time points (*p* > 0.05) (Supplementary Figure S1).

### 2.2. The Combination of ACEi + ARB Improves Respiratory Capacity

**Figure 2:**
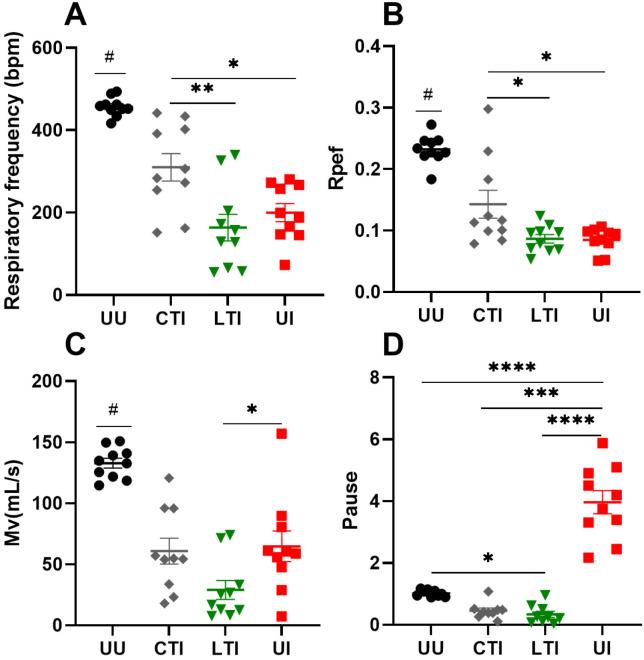
Assessment of lung capacity through the parameters of respiratory frequency (**A**), Rpef (**B**), minute volume (Mv) (**C**), and Pause (**D**) in uninfected control or SARS-CoV-2 infected K18-hACE2 mice treated or not with Losartan or Losartan + Lisinopril combination at 6/7 days post-infection. UU = Untreated Uninfected, CTI = Combination-treated Infected, LTI = Losartan-treated Infected, and UI = Untreated Infected. The normality of the data was assessed using the Shapiro–Wilk test. Data of (**A**–**C**) showed normal distribution and were analyzed using a parametric test, one-way ANOVA followed by the Bonferroni post-test. Data of (**D**) did not show normal distribution, and a non-parametric analysis was performed, with the Kruskal–Wallis test followed by the Dunn post-test. (#) Significant difference, all groups versus the UU group. (*) Significant difference between specific groups as indicated by the lines: * *p* < 0.05, ** *p* < 0.005, *** *p* < 0.0005 and **** *p* < 0.0001. *n* = 10 per group.

Lung capacity was assessed at 6/7 dpi using parameters such as respiratory frequency (RF), the ratio of peak expiratory flow to total expiratory time (Rpef), the rate of ventilation or minute volume (Mv), and respiratory pause (Pause). All infected groups demonstrated significant reductions in respiratory frequency, Rpef, and minute volume compared to the Untreated Uninfected group (padj ≤ 0.005) (Figure 2A–C). However, the infected group treated with combination therapy exhibited significantly better performance in both respiratory frequency and Rpef than the Untreated Infected and the Losartan-treated Infected groups. Additionally, Untreated Infected mice showed a significant increase in the pause parameter, which was prevented by both Losartan and combination therapy (Figure 2D). These findings suggest that the combination of Lisinopril and Losartan substantially enhances respiratory capacity in infected mice. No sex-based differences were observed across any analyzed groups (*p ≥* 0.05) (Supplementary Figure S2).

### 2.3. Treatments Modulate Lung ACE2 Levels, the RAS System, and SARS-CoV-2 Pulmonary Load

**Figure 3:**
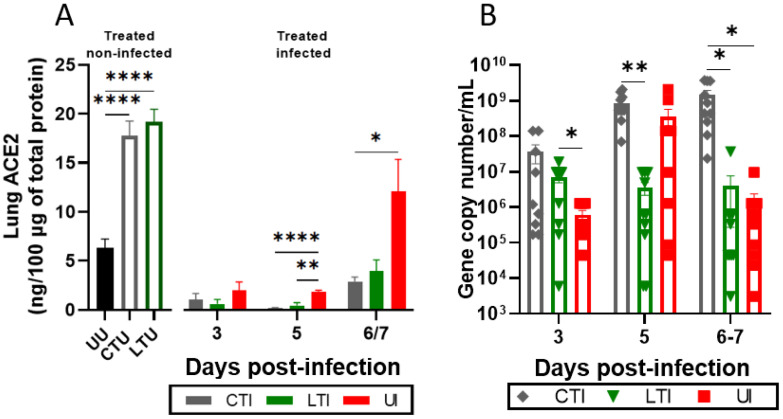
(**A**) Quantification of ACE2, on lungs, by ELISA in uninfected mice treated or not with Losartan or Losartan + Lisinopril combination over 21 days (black and white columns; UU: uninfected untreated; CTU: combination-treated, uninfected; LTU: Losartan-treated, uninfected) and in the same animals after infection with SARS-CoV-2 (colored columns; CTI [gray]: combination-treated, infected; LTI [green]: Losartan-treated, infected; UI [red]: untreated, infected). (**B**) Viral load in SARS-CoV-2-infected K18-hACE2 mice treated or not with Losartan or Losartan-Lisinopril combination on days 3, 5, or 6/7 post-infection. (*) Significant difference between groups: * *p* < 0.05, ** *p* < 0.005, **** *p* < 0.0001. The normality of the data was assessed using the Shapiro–Wilk test. A one-way ANOVA was performed, followed by the Bonferroni post-test (A-Treated non-infected), and mixed-effects analyses were conducted, followed by the Tukey post-test (A-Treated infected/B). (*) Significant difference between specific groups as indicated by the lines: * *p* < 0.05, ** *p* < 0.005, **** *p* < 0.0001. Data are presented as mean ± SEM. *n* = 10 per time point.

K18-hACE2 mice were treated with either the combination of Lisinopril + Losartan (CTU) or Losartan alone (LTU) for 21 days without infection to investigate the impact of these treatments on ACE2 modulation. Figure 3A illustrates that both Losartan and combination therapy significantly increased pulmonary ACE2 levels compared to Untreated Uninfected animals (*p* < 0.0001). SARS-CoV-2 infection led to a significant reduction in lung ACE2 levels at 3 and 5 dpi, regardless of treatment, with a rebound in ACE2 levels at 6/7 dpi, most notably in the Untreated Infected group (Figure 3A). No sex-based differences were observed (*p* > 0.05) (Supplementary Figure S3A).

In the untreated groups, SARS-CoV-2 viral loads were detected in the lungs at 3 dpi, with an increase at 5 dpi, followed by a decrease at 6/7 dpi (Figure 3B). Treatment with either Losartan or Losartan + Lisinopril combination therapy resulted in increased lung viral loads at 3 dpi. However, Losartan-only therapy effectively prevented further increases in viral load at 5 and 6/7 dpi, whereas combination therapy led to a sustained increase in viral load, remaining significantly higher at 6/7 dpi compared to Untreated Infected mice (*p* < 0.005). Differences between sexes were observed only in the CTI group at 3 dpi, with no significant differences observed in the other group’s sexes (*p* > 0.05) (Supplementary Figure S3).

### 2.4. Losartan and Lisinopril + Losartan Combination Therapies Downregulate the Inflammatory Response During SARS-CoV-2 Infection

**Figure 4:**
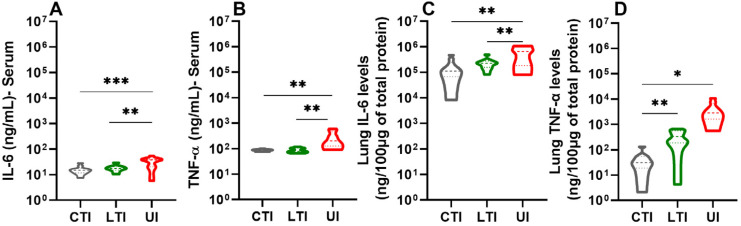
Analysis of IL-6 and TNF-α in serum (**A**,**B**) and lung (**C**,**D**) of SARS-CoV-2-infected K18-hACE2 mice treated or not with Losartan or Losartan + Lisinopril combination at 6/7 dpi. CTI = Combination-treated Infected, LTI = Losartan-treated Infected, and UI = Untreated Infected. (*) Significant difference: * *p* < 0.05, ** *p* < 0.005 and *** *p* < 0.0005. Analysis was performed using ANOVA, followed by Bonferroni’s multiple comparisons test. Data presented as mean ± SEM. *n* = 10 mice per group.

The levels of pro-inflammatory cytokines IL-6 and TNF-α, which are key indicators of COVID-19 severity, were quantified in serum and lung tissue at 6/7 dpi. The Untreated Infected group exhibited significantly elevated levels of cytokines in the serum and lungs. However, either treatment (Losartan or the combination therapy) led to a marked reduction in cytokine levels (*p* < 0.005) (Figure 4). No significant sex-based differences were observed across the groups (Supplementary Figure S4).

### 2.5. Losartan Treatment Decreases the Severity of Histopathological Findings

**Figure 5:**
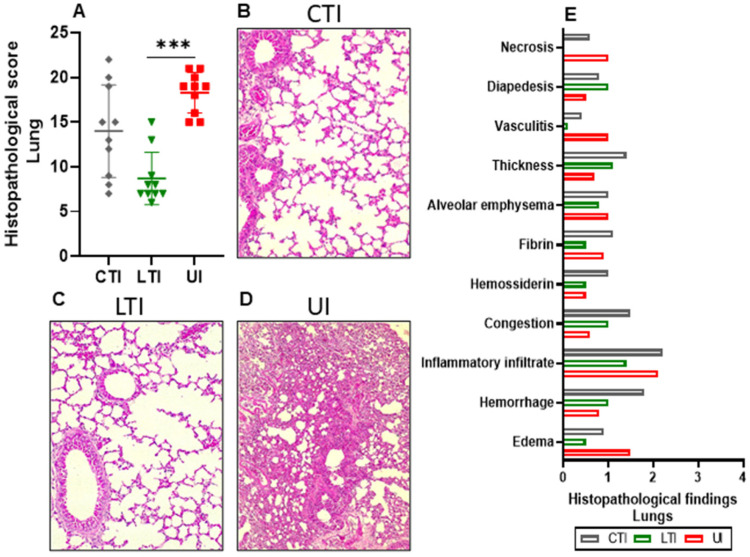
Histopathological analysis of lungs at days 6/7 post-infection of SARS-CoV-2-infected K18-hACE2 mice treated of not with Losartan or Losartan-Lisinopril combination. CTI = Combination-treated Infected, LTI = Losartan-treated Infected, and UI = Untreated Infected. (**A**) Results obtained from the sum of the degrees of intensity (maximum score of 44), *** *p* < 0.0005. Data are presented as individual scores and mean ± SD. The normality was assessed using the Shapiro–Wilk test. The Kruskal–Wallis test was used, followed by the Dunn post-test. (**B**–**D**) Representative histopathological images, emphasizing inflammatory infiltrate and thickness: (**B**) CTI-mice (score 1), (**C**) LTI-mice (score 1), and (**D**) UI-mice (score 4). (**E**) Histopathological findings, data presented as mean. *n* = 10 animals per group. Bar = 50 µm.

Untreated Infected mice significantly presented several histopathological changes in the lungs at 6/7 dpi, resulting in a high combined histopathological score of ~19 (Figure 5A). In contrast, Losartan treatment led to a substantial reduction in the histopathological score (~8) at 6/7 dpi, compared to the Untreated Infected group (*p* = 0.0002). However, the combination-treated infection group exhibited considerable variability in the results, and consequently, no significant difference in histopathological scores was observed when compared to other experimental groups (Figure 5A–D).

The frequency of histopathological findings also varied across the groups. Inflammatory infiltrates were presented in all treated and infected samples, and 90% (9/10) of the Untreated Infected group. Necrosis was absent in the Losartan-treated Infected group but observed in 40% (4/10) of the slides from both the Combination-treated Infected and Untreated Infected groups. Similarly, vasculitis was identified in only 10% (1/10) of the Losartan-treated Infected group, whereas it was recorded in 70% (7/10) of the Combination-treated Infected and Untreated Infected groups. Interestingly, no severe or high-intensity histopathological findings were observed in the Losartan-treated Infected group (Figure 5E). No significant sex-based differences were noted (*p* > 0.05) (Supplementary Figure S5).

### 2.6. Treatment Does Not Impact the Mortality Rate but Improves Clinical Outcomes

**Figure 6:**
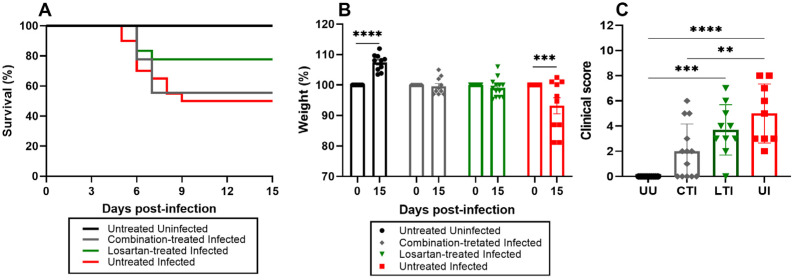
(**A**) Survival curve of uninfected control (black line, *n* = 10) and SARS-CoV-2-infected K18-hACE2 mice untreated (red line, *n* = 20) or treated with Losartan (green line, *n* = 18) or Losartan-Lisinopril combination (gray line, *n* = 18). Untreated Uninfected did not show mortality. No significant differences in survival were observed between infected groups. The survival log-rank (Mantel–Cox) test was applied. (**B**) Analysis of weight loss of uninfected controls (black columns) or survivor SARS-CoV-2-infected K18-hACE2 mice untreated (red columns) or treated with Losartan (green columns) or Losartan-lisinopril (gray columns) at day 0 (pre-infection) and 15 days post-infection (dpi). (**C**) clinical scores of the experimental groups at 15 dpi. Normality was assessed using the Shapiro–Wilk test. Analysis was performed using ANOVA, followed by Bonferroni’s multiple comparisons test. (*) Significant differences: ** *p* < 0.005, *** *p* < 0.0005 and **** *p* < 0.0001.

Survival analyses revealed no significant differences in mortality rates between the treated Infected groups and the Untreated Infected group (Figure 6A). Although the Losartan-treated Infected group exhibited a trend toward lower mortality, this difference did not reach statistical significance (*p* = 0.1214). At 15 dpi, body weight increased by 8.0 ± 5.24% in Uninfected control animals, while among survivors in the Untreated Infected group, body weight decreased by 8.0 ± 4.80% compared to their baseline weight at the time of infection with SARS-CoV-2 (Figure 6B). Mice treated with either Losartan or the Losartan + Lisinopril combination therapy showed no significant changes in body weight at 15 dpi. Notably, while all infected mice (treated and untreated) experienced weight loss of 10–20% by 7 dpi, all survivors showed some recovery by 15 dpi, with the treated groups exhibiting more prominent recovery. Regarding clinical scores at 15 dpi, all groups demonstrated clinical improvement, though persistent manifestations were still observed. The combination therapy group exhibited significantly better clinical outcomes compared to other groups (Figure 1 and Figure 6B,C). These clinical findings indicate that while treatments did not affect mortality rates, they significantly improved clinical recovery in infected mice.

## 3. Discussion

During the early stages of the COVID-19 pandemic, there was considerable debate regarding the use of antihypertensive medications, particularly ACEis and ARBs, in patients infected with SARS-CoV-2. This concern stemmed from the dual role of ACE2, which is involved in both viral replication and regulation of the RAS mechanisms. The potential for ACEi and/or ARB use to increase ACE2 expression raised concerns about the possible exacerbation of viral load and the development of more severe forms of COVID-19 [13,32,33], as higher ACE2 levels could theoretically enhance viral load and worsen disease outcomes. The purpose of this study was to examine the effects of ACEi and ARB use on viral load and relevant outcome measures in controlled experiments.

The major findings of this study can be summarized as follows. As previously observed with Lisinopril [31], a 21-day treatment regimen with Losartan or Losartan + Lisinopril combination led to marked increases in ACE2 levels in the lungs of K18-hACE2 mice. Upon infection with SARS-CoV-2 (Wuhan strain), treated mice showed higher viral loads at 3 dpi. However, the higher viral loads did not result in worse outcomes; on the contrary, both Losartan- and Losartan + Lisinopril-treated mice showed overall better clinical scores, body weight, respiratory function and lung histopathological scores, and although survival rate was not significantly affected, survivors in the treated groups recovered better than those in the untreated group. In general, mice in the Losartan-Lisinopril combination therapy showed better performances in the above parameters than mice in the Losartan-only group. The benefits observed in the treated groups were associated with a substantial decrease in the levels of the pro-inflammatory cytokines IL-6 and TNF-α, which are key indicators of COVID-19 severity.

The increased lung ACE2 expression after treatment with Lisinopril, Losartan, or Combined therapy is consistent in different studies [12,31]. Some studies reported that Lisinopril can promote a 5-fold increase in ACE2 levels, while Losartan increases it by 3-fold [13,34,35]. Our data indicate similar potency (about 3-fold) of Lisinopril, Losartan, or the Combination in increasing lung ACE2 levels. Lisinopril reduces the conversion of angiotensin I to angiotensin II, reducing the vasoconstrictive and inflammatory effects of angiotensin II. Meanwhile, Losartan blocks the AT1R, preventing the adverse effects of its activation [36,37].

SARS-CoV-2 infection led to a sharp decrease in lung ACE2 levels at 3 dpi. This reduction likely reflects ACE2 consumption following virus binding and internalization via spike protein. This effect is also observed in animals treated with Lisinopril [31], Losartan, or their combination. And since Lisinopril and/or Losartan-treated mice have much higher pre-infection levels of ACE2, its reduction at 3 dpi was even sharper in these animals. Also, this implies that a greater number of viral particles are expected to be internalized and replicated in the treated animals, which effectively happened, as Losartan- and/or Lisinopril-treated animals showed higher lung viral loads. These data indicate that higher ACE2 density at baseline is linked with higher infection incidence or efficiency.

Accordingly, the hypothesis that ACE2 levels influence COVID-19 severity has been explored, with different studies showing contrasting results. Some clinical studies have indicated that higher levels of soluble circulating ACE2 (sACE2), either at baseline (admission) or increasing during hospitalization, were associated with disease severity or mortality [38,39,40]. These associations could be interpreted in the sense that higher ACE2 levels would lead to higher viral loads and, therefore, to more severe disease, since clinical studies have shown associations of higher viral load with disease severity, ICU hospitalization, and mortality [41]. In contrast, other studies show the opposite, with lower levels of sACE2 associated with worse outcomes [42,43,44]. In these clinical studies, sACE2 is taken as a measure of ACE2 shed from cell membranes in different organs; measures of ACE2 levels in specific organs such as the lungs are not available.

In the present and in a prior study, treatment with Losartan only and Losartan + Lisinopril, as well as with Lisinopril only [31], led to a marked increase in ACE2 levels in the lungs of mice. And treated mice with higher baseline ACE2 levels showed greater viral loads during infection. Interestingly, the increase in viral load was persistent for animals treated with Lisinopril only and Lisinopril + Losartan combination, but not with Losartan only. Despite the higher viral load, a more severe outcome of the infection was not observed. The treated mice actually performed better in clinical scores, body weight measurements, and histopathological changes than the untreated mice.

This outcome aligns with findings from clinical studies, where ACEi + ARB was not associated with the severity of COVID-19 or worsening clinical conditions, increased length of hospital stay, or mortality rate. On the contrary, the use of ACEis and/or ARBs reduced the number of intensive care unit admissions in hypertensive patients [27,45,46]. Additionally, these medications have been linked to reduced D-dimer levels and pro-inflammatory cytokines, indicating a potential protective effect [47,48,49,50,51,52]. In alignment with these findings, a recent study by de Sá et al. (2024) [53] highlighted the critical balance between viral replication and pulmonary inflammation in lethal cases of COVID-19. The study found that patients with higher viral loads exhibited reduced inflammatory responses, suggesting that ACE2 modulation played a key role in their outcomes [53].

The favorable outcomes observed in the present study can be explained as Losartan-only and Losartan + Lisinopril combination treatments resulted in marked reductions in the levels of the pro-inflammatory cytokines IL-6 and TNF-α following infection by SARS-CoV-2. Similar effects were observed with Lisinopril-only therapy in experimental COVID-19 [31] and symptomatic patients treated with Losartan in a multicenter study [54]. Meng and colleagues [24] hypothesized that ACE inhibitors (ACEis) do not directly inhibit viral replication but modulate immune function and suppress inflammatory responses.

Factors beyond a diminished inflammatory response may contribute to the benefit observed in SARS-CoV-2-infected mice treated with Losartan, Lisinopril, or Combination therapy. ACE2, a critical regulator of the renin–angiotensin system, plays a significant role in COVID-19 pathogenesis. COVID-19 can be considered a severe vasculopathy, causing vascular activation, dysfunction, vasoconstriction, and thrombotic events [9,55]. Therefore, treatments with Losartan and/or Lisinopril, in addition to mitigating the pro-inflammatory response, may have also affected vascular and thrombotic complications. Further studies are necessary to establish these potential benefits.

All infected groups exhibited reduced lung capacity measured by whole-body plethysmography, including decreased RF, Rpef, and tidal volume, as observed in previous experimental COVID-19 studies [56,57,58]. However, mice treated with the Losartan + Lisinopril combination, but not Losartan alone, showed better lung function, with improved performances in most parameters.

Interestingly, Losartan-only-treated mice showed lower viral loads in the lungs during infection, which may help to explain the similar clinical outcomes observed in the Losartan and Losartan + Lisinopril groups. Both treatment groups performed better in clinical scores, body weight retention, histopathological assessments, and overall disease progression compared to Untreated Infected animals.

The use of an alternative ARBs could lead to improved mortality outcomes. Comparative studies between Telmisartan and Losartan in hospitalized COVID-19 patients have highlighted the chemical and pharmacological differences that may influence therapeutic efficacy. Telmisartan is more lipophilic, facilitating penetration into tissues and cells. This may explain its direct pulmonary benefits, such as reduced use of mechanical ventilation and the need for supplemental oxygen, along with lower mortality and ICU admissions [59,60]. An open multicenter clinical trial showed that Telmisartan significantly reduced mortality and accelerated hospital discharge in COVID-19 patients, showing an 81% reduction in the risk of death compared to other recommended treatments [61]. Additionally, innovative research is exploring the potential of Telmisartan in advanced formulations, such as nanosuspensions for inhaled therapy, to treat lung diseases and other respiratory infections [62].

After 15 days of observation, survivor mice in the treated groups completely recovered, including weight restoration, regression of clinical manifestations, and a return to normal behavior. Overall, these results indicate that Losartan or the combination of Losartan + Lisinopril treatments provide significant benefits by decreasing clinical and pathological manifestations during the acute phase of experimental COVID-19 and facilitating the recovery of survivor animals.

This study has several limitations. A single SARS-CoV-2 strain (Wuhan) was used, which, while ensuring consistency in the results, does not account for potential variations in disease severity and immune response across different viral variants [63]. Future studies incorporating multiple SARS-CoV-2 variants would provide a broader understanding of how ACEi/ARB treatments influence disease progression in diverse viral backgrounds. Also, this study was conducted with healthy, young animals (10–12 week old mice), which received treatment for 21 days before infection. The use of ACEis/ARBs is mainly by people with chronic conditions such as hypertension, usually at older ages and many times with multiple comorbidities, which are risk factors for severe COVID-19 [11,15]. The use of animals in conditions that better mimic these features, and treated for longer periods, could potentially reflect more accurately the scenarios where humans are affected by the disease. Additionally, the incidence and time for development of critical respiratory dysfunction in this model is variable, with about 50% of the SARS-CoV-2-infected animals succumbing between 5 and 9 dpi [31,56]. For this reason, the analyses, including histopathology, performed on 6 and 7 dpi include mice at different stages of disease and variability, is therefore expected. Finally, this study focused solely on the acute phase of the disease. The long COVID-19 syndrome has been recognized as prolonged, persistent complications of the disease [64,65,66], and extended follow-up study designs aimed at determining the effects of ACEis/ARBs on long COVID-19 are needed.

In conclusion, our results indicate that the treatment with Losartan alone or the Losartan–Lisinopril combination contributed to the clinical improvement of these animals, exhibiting reduced body weight loss, lower clinical scores, improved lung capacity, lesser histopathological lung damage, and milder inflammatory responses compared to the Untreated Infected group. However, treatment did not significantly improve survival. These findings provide further evidence for the beneficial role of ACEi/ARB therapies in managing COVID-19, potentially improving patient outcomes by attenuating inflammation and balancing replication.

## 4. Materials and Methods

### 4.1. Experimental Model and Ethics

The research employed the B6.Cg-Tg (K18-hACE2) 2Prlmn/J strain 034,860 animal model, which has a C57BL/6 genetic background and expresses human ACE2 (hACE2) through a cytokeratin 18 (K18) promoter, predominantly in the epithelial cells of the respiratory airways. [67,68,69]. These mice were generously supplied by the Institute of Science and Technology in Health of the Oswaldo Cruz Foundation (ICTB-FIOCRUZ), Rio de Janeiro, Brazil. The study received ethical approval from the Ethics Committees for the Use of Laboratory Animals of the Faculty of Pharmaceutical Sciences/University of São Paulo (FCF/USP) and the Institute of Biomedical Sciences/USP (ICB/USP), with license numbers 622 and 3858020621, respectively. All procedures were conducted in strict adherence to ethical guidelines and biosafety protocols, in compliance with Federal Law 11.794/2008 and the National Council for Animal Experimentation (CONCEA). The Internal Biosafety Committee (CIBIO-0092021/FCF) approved this study. Activities involving viral manipulation, animal infection, and sample collection were conducted within a Biosafety Level 3 Laboratory (BSL3).

### 4.2. Treatment

Losartan (Aché Corus-Brazil registration 1121301790102) or the combination of Losartan and Lisinopril (Medley–Brazil registration 101810399008-7) were administered continuously for 21 days; then mice were infected with SARS-CoV-2 and treatment was extended for the duration of infection (up to seven days post-infection in most experiments and up to 15 days in the survival experiment). Mice were allocated into the following groups: Untreated Uninfected (UU), Combination-treated Uninfected (CTU), Losartan-treated Uninfected (LTU), Untreated Infected (UI), Combination-treated Infected (CTI), and Losartan-treated Infected (LTI). Losartan, with or without Lisinopril, was incorporated into flavored gelatin to improve palatability and encourage voluntary consumption. Each day, mice received a dose of 10 mg/kg/day, while those in the UU and UI groups were given gelatin without medication.

### 4.3. Virus, Infection, and Experimental Design

After 21 days of treatment with Losartan or the combination of Losartan and Lisinopril, the mice were infected following a previously established protocol [56]. The SARS-CoV-2 virus, sourced from Professor Edison Durigon’s team at the Department of Microbiology, ICB/USP, was propagated according to the methodology described by Araújo et al. [70]. Mice were lightly sedated with isoflurane and intranasally infected with 10^5^ plaque-forming units (PFU) of SARS-CoV-2 (original Wuhan strain: SARS.CoV2/SP02.2020.HIAE.Br). Mice were checked daily for clinical scores and body weight. For determination of viral load and lung ACE2 expression, groups of mice were subjected to euthanasia on 3, 5, and 6/7 dpi, and organs and blood were harvested. Respiratory function was measured on 6/7 days post-infection (dpi). The notation “6/7 dpi” refers to the fact that, specifically on 6 dpi, measurements and euthanasia were conducted on animals that reached the humane endpoint as described in Section 4.5, and, on 7 dpi, measurements and euthanasia were conducted on all remaining animals. The 6/7 dpi timepoint was chosen as it was previously determined that this is a critical phase of infection, with SARS-CoV-2-infected mice displaying severe respiratory dysfunction and mortality occurring between 5 and 9 dpi [31]. Specifically, at 6 dpi, measurements were conducted on animals that reached the humane endpoint as described in Section 4.5 and, at 7 dpi, measurements were conducted on all remaining animals. Lung histopathology and cytokine levels were determined on 6/7 dpi as well.

### 4.4. Assessment of Lung Capacity

To assess the respiratory pattern, mice were placed in plethysmographic chambers (BUXCO Electronics, Wilmington/NC/ USA) on days 6/7 post-infection. Whole-body plethysmography (WBP), a non-invasive method, was employed to evaluate the respiratory capacity of non-anesthetized animals by recording pressure changes reflected in waves proportional to respiratory flow, without movement restrictions, for 10 min. The equipment measured various parameters, including respiratory frequency (RF), Minute volume (Mv), respiratory pause (Pause), and the ratio of peak expiratory flow to total expiratory time (Rpef). RF denotes the number of complete breaths per minute. Pause is calculated from the expiratory time (Te) under 65% of the air volume expiration time (Rt), minus 1 (Pause = Te/Rt − 1). Rpef, akin to Pause, is an indicator of constriction and is calculated as the ratio between peak expiratory flow (PEF) and Te [57,71,72,73].

### 4.5. Clinical Evaluation

The mice underwent daily monitoring, during which behavioral changes were systematically observed, including lethargy, labored breathing, hunched posture, piloerection, tremors, ocular and nasal exudate, eye closure, and mortality. These symptoms were recorded in an individual score table, where a score from 0 to 4 was assigned to represent the severity of the manifestation (0 represents no clinical signs, 1 indicates mild signs, and 4 signifies severe signs). This scoring system and table were developed in accordance with the guidelines set forth in the Humanitarian Endpoint Implementation Guide of the Federal University of São Paulo [74]. Additionally, signs of pain were monitored using the Grimace Scale for mice [75]. A humane endpoint was established based on criteria such as weight loss (<20%), animal inactivity, and lack of response to external stimuli.

### 4.6. Euthanasia and Sample Collection

The animals were euthanized through intraperitoneal administration of an anesthetic combination of Ketamine (150 mg/Kg) and Xylazine (15 mg/Kg), followed by exsanguination via cardiac puncture for blood collection. Subsequently, cardiac perfusion was conducted with PBS, adhering to the previously described protocol [76]. Samples intended for viral quantification via qRT-PCR were preserved in RNA later (Invitrogen-Cat.7021, Waltham, MA, USA). For enzyme-linked immunosorbent assay (ELISA) analysis, samples were preserved in radioimmunoprecipitation assay (RIPA) buffer (Thermo Fisher-Cat.-89900, Waltham, MA, USA) supplemented with protease inhibitors (Sigma-Aldrich Cat.S8830-20TAB, Saint Louis, MO, USA). All biological samples were promptly frozen and maintained at −80 °C. Tissue fragments for histopathology analysis were collected in 10% formaldehyde and preserved in 70% ethanol until further processing.

### 4.7. Protein Extraction

The tissue samples were finely macerated and subsequently resuspended in RIPA buffer (Thermo Fisher Cat. 89900, Waltham, MA, USA) containing a protease inhibitor cocktail (Sigma-Aldrich-Cat. 58830, Saint Louis, MO, USA). They were centrifuged at 2000 rotations per minute (rpm) for 5 min at 4 °C, and the resulting supernatants were transferred to new 1.5 mL microtubes. A second centrifugation was performed at 12,000 rpm for 20 min, and then supernatants were transferred to new microtubes and stored at −80 °C until processing. Total protein concentration in each sample was quantified using the Pierce BCA protein assay kit (Thermo Fisher-Cat. 23225, Waltham, MA, USA), following the manufacturer’s instructions. Protein quantification was performed using a Multiskan™ FC Microplate Photometer (Thermo Fisher-Cat. 51119000, Waltham, MA, USA).

### 4.8. Quantification of ACE2 in Tissue Samples by ELISA

Detection and quantification of ACE2 protein in tissue samples were performed using the Human ACE2 DuoSet ELISA kit (R&D Systems- Cat. DY933-05, Minnneapolis, MN, USA), following the manufacturer’s recommendations. ELISA plates were coated overnight at room temperature (RT) with 100 µL of anti-ACE2 antibody (2 µg/mL, diluted in PBS, pH 7.4). Following coating, the plates were washed three times with 300 µL of washing buffer (PBS + 0.05% Tween 20) and blocked with 300 µL of blocking buffer (1% BSA in PBS, pH 7.4) for 1 h at RT. After blocking, the plates were washed again three times, and 100 µL of standards or tissue samples were added to each well. In some instances, the samples were diluted in dilution reagent (and their respective correction factors were assigned in the result analysis) provided by the kit and incubated for 1 h at RT. Then, the plates were washed, and 100 µL of detection antibody (100 ng/mL, diluted in 1% BSA in PBS, pH 7.4) was added and incubated for 1 h. After another washing step, 100 µL of secondary antibody (conjugated with HRP) was added to each well for one hour at RT. Subsequently, the plates were washed again, and 100 µL of TMB substrate was added to the wells. The colorimetric reaction was stopped by adding 50 µL of 1 M sulfuric acid, and absorbance was measured at 450 nm using a microplate reader. Protein quantification was performed using a Multiskan™ FC Microplate Photometer (Thermo Fisher-Cat. 51119000, Waltham, MA, USA).

### 4.9. RNA Extraction and Virus Detection

Lung tissues were homogenized with MagNA Lyser equipment (Roche Diagnostics, Mannheim, Germany) and subsequently centrifuged at maximum speed for 15 min. A 150 μL aliquot of the supernatant was mixed with 200 μL of Lysis Buffer (BioMerieux, Lyon, France). Then, total nucleic acid extraction was performed according to the protocol established by the Nuclisens MagMax extraction system (BioMerieux, Lyon, France).

The extracted genetic material was subjected to RT-qPCR for the detection of SARS-CoV-2 and mammalian ribonucleoprotein (RNP), which served as an extraction controls. The AgPath-ID™ One-Step RT-PCR kit (Applied Biosystems/Life Technologies, Austin, TX, USA) and 7500 Real-Time Systems equipment (Applied Biosystems/Life Technologies, Foster City, CA, USA) were used, with protocols adapted from Corman et al. [77]. The forward, reverse, and probe primers targeting the SARS-CoV-2 spike protein and RNP were used in equal proportions and concentrations of 10µM. Negative and positive controls included Nuclease Free Water and a clinical isolate in Vero-E6 cells respectively, both of which were rigorously, tested and standardized. The RT-qPCR amplification conditions were as follows [77]: 45 °C for 15 min, once; 95 °C for 10 min, once; 95 °C for 15 s, and 57 °C for 1 min, 45 times. Following viral detection via RT-qPCR, the viral load of each sample was quantified in terms of copies/mL.

### 4.10. Cytokine Quantification

Levels of IL-6 (Thermo Fisher-Cat.88-7064-88, Waltham, MA, USA) and TNF-α (Thermo Fisher-Cat. 88-7324-88, Waltham, MA, USA) were quantified in serum and lung tissue samples at the study endpoint, following the manufacturer’s instructions (Thermo Fisher-Cat.88-7064-88, Waltham, MA, USA). ELISA plates were coated with 100 µL of IL-6 or TNF-α standard and incubated overnight. Afterward, at RT, the plates underwent three washes with 300 µL of washing buffer, followed by blocking with 1% BSA in PBS (pH 7.4) for 1 h at RT. Subsequently, 100 µL of either standard or sample was added to each well. In some cases, sample dilutions were using the provided dilution reagent (with respective correction factors assigned for result analysis) and incubated for 1 h at RT. Following this, the plates were rewashed, and 100 µL of detection antibody was added to each well for an additional hour of incubation at RT. Following a final wash step, 100 µL of secondary antibody HRP-conjugated was added, and the plates were incubated for one hour at RT. Subsequently, the plates were washed, and 100 µL of TMB substrate was added to each well. The colorimetric reaction was halted by adding 100 µL of 1 M sulfuric acid, and the absorbance was measured using a microplate reader to quantify the cytokine levels in the samples at 450 nm (Thermo Fisher-Cat. 51119000, Waltham, MA, USA).

### 4.11. Survival

Mice were treated with Losartan or a combination of Losartan + Lisinopril for 21 days via flavored gelatin, and were then infected with 10^5^ PFU of SARS-CoV-2. The treatment protocol continued for an additional 15 days via oral gavage. The Untreated Uninfected group received flavored gelatin without any medication, and, following infection, received PBS via oral gavage. Weight loss and clinical manifestations were meticulously monitored in accordance to the humane endpoint criteria detailed in Section 4.5.

### 4.12. Statistical Analysis

The data were systematically entered into a database using Excel software and analyzed individually using appropriate statistical methods tailored to the specific experimental design. The normality of the data was checked using Kolmogorov–Smirnov, D’Agostino–Pearson, and Shapiro–Wilk tests. For parametric variables, statistical analysis was performed using the Student’s t-test or multiple T-tests, as well as ANOVA followed by Bonferroni’s post hoc test for multiple comparisons. Non-parametric data were analyzed using the Mann–Whitney U test and the Kruskal–Wallis tests, with Dunn’s multiple comparisons test. Survival curves were compared using the log–rank (Mantel–Cox) test. Statistical significance was defined as *p*-value < 0.05. Graph Prism software version 8.0 was employed for all analyses.

## Data Availability

The original contributions presented in this study are included in the article/Supplementary Material. Further inquiries can be directed to the corresponding author(s).

## Supplementary Materials

The following supporting information can be downloaded at: https://www.mdpi.com/article/10.3390/ijms26167663/s1.
