# Supplementary figures and images for: Angiotensin-Converting Enzyme Inhibition and/or Angiotensin Receptor Blockade Modulate Cytokine Profiles and Improve Clinical Outcomes in Experimental COVID-19 Infection
Source: Int J Mol Sci. 2025 Aug 8;26(16):7663. doi: 10.3390/ijms26167663 (PMC12387000; doi:10.3390/ijms26167663)

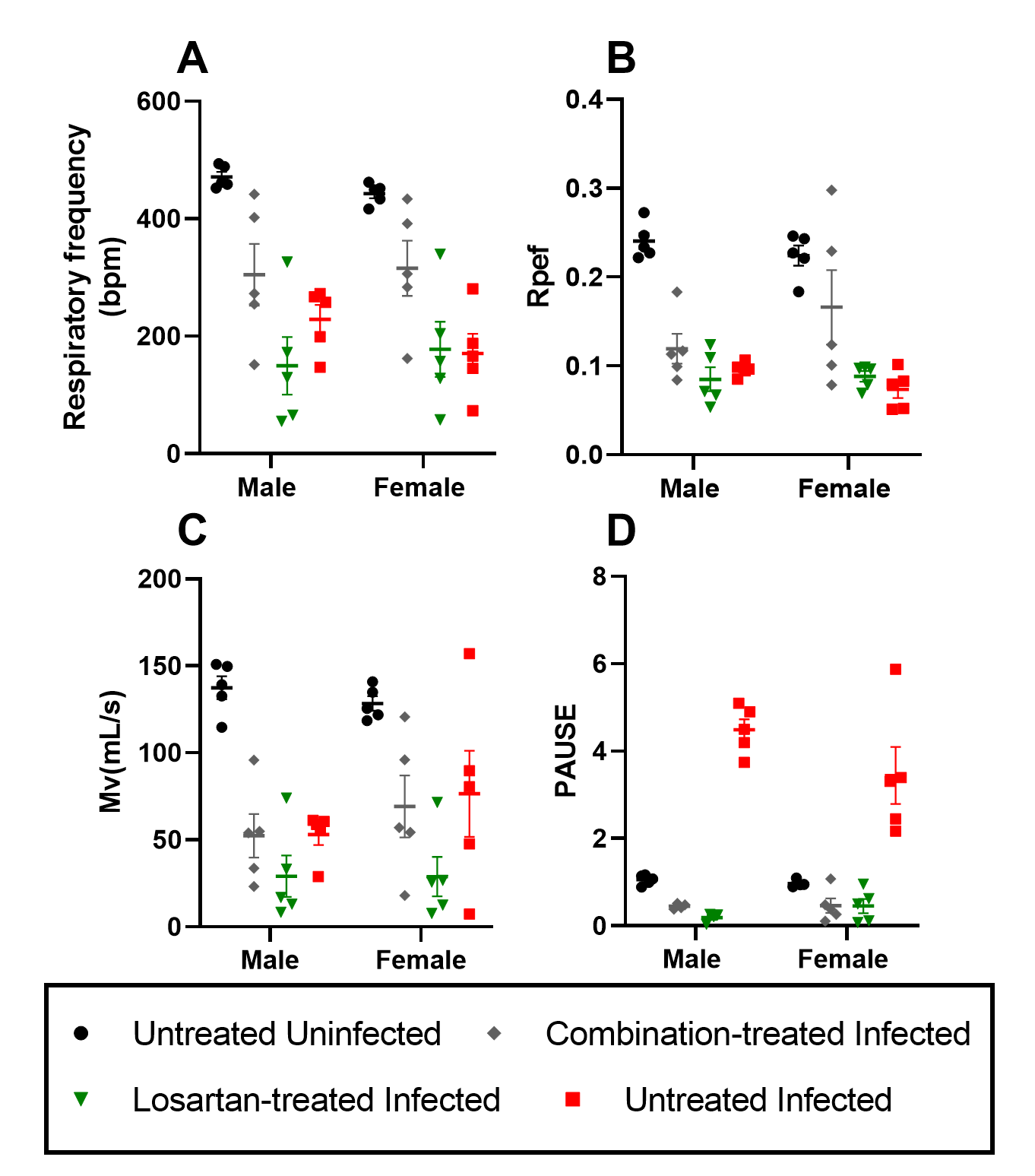

Supplement: Supplementary file 1 [file ijms-26-07663-s001.zip › SupFigure S2.tif]

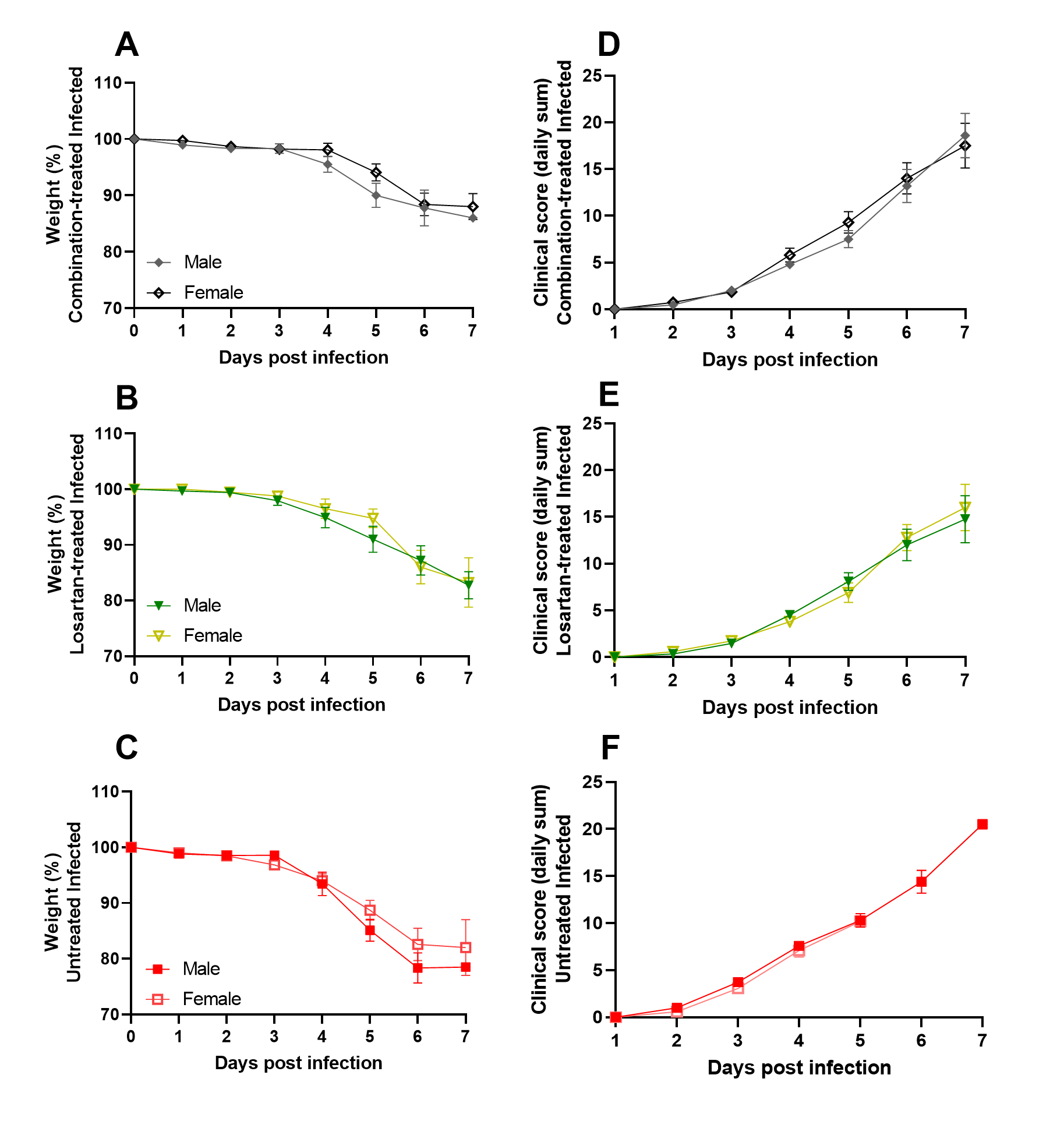

Supplement: Supplementary file 1 [file ijms-26-07663-s001.zip › SupFigureS1.tif]

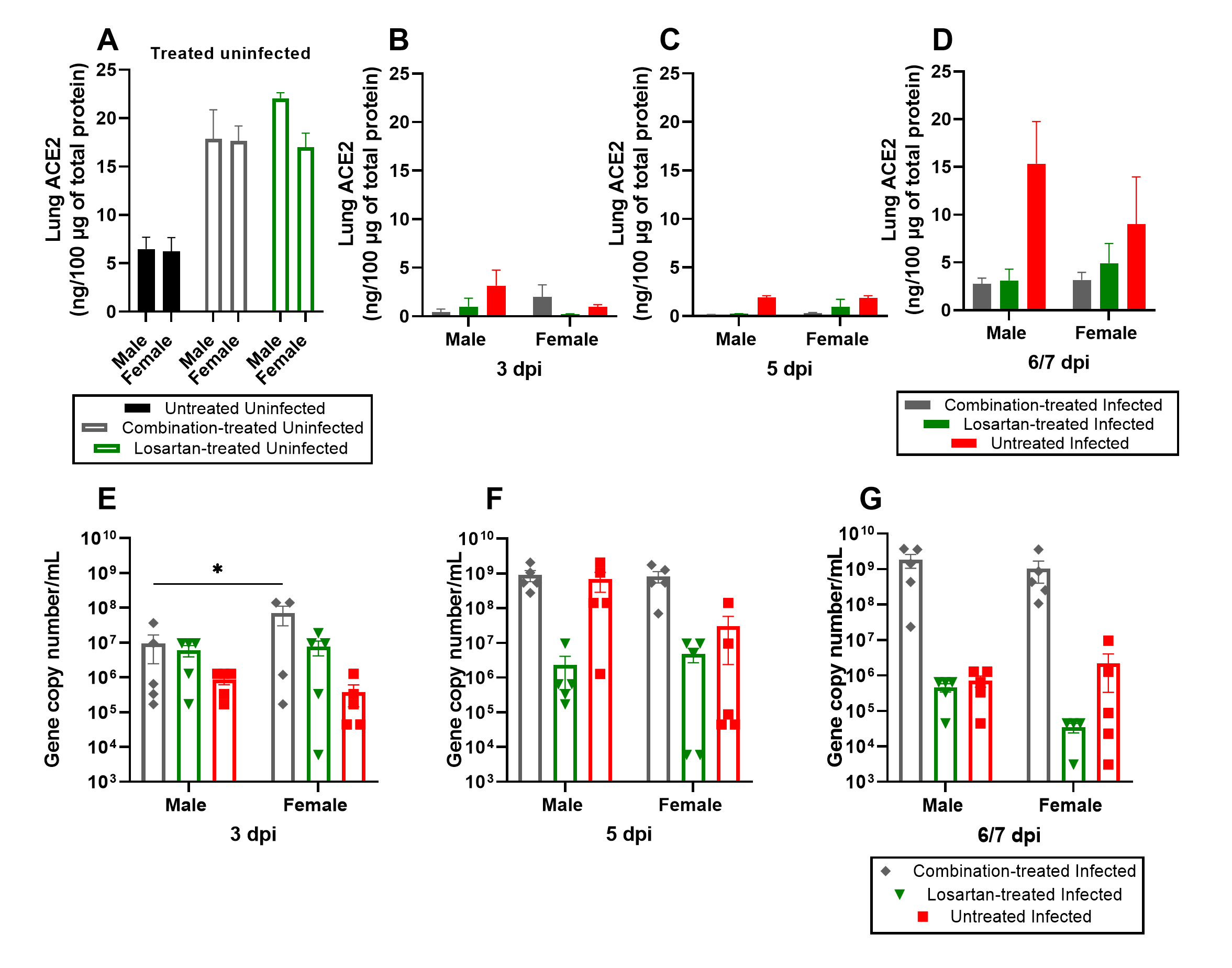

Supplement: Supplementary file 1 [file ijms-26-07663-s001.zip › SupFigureS3.tif]

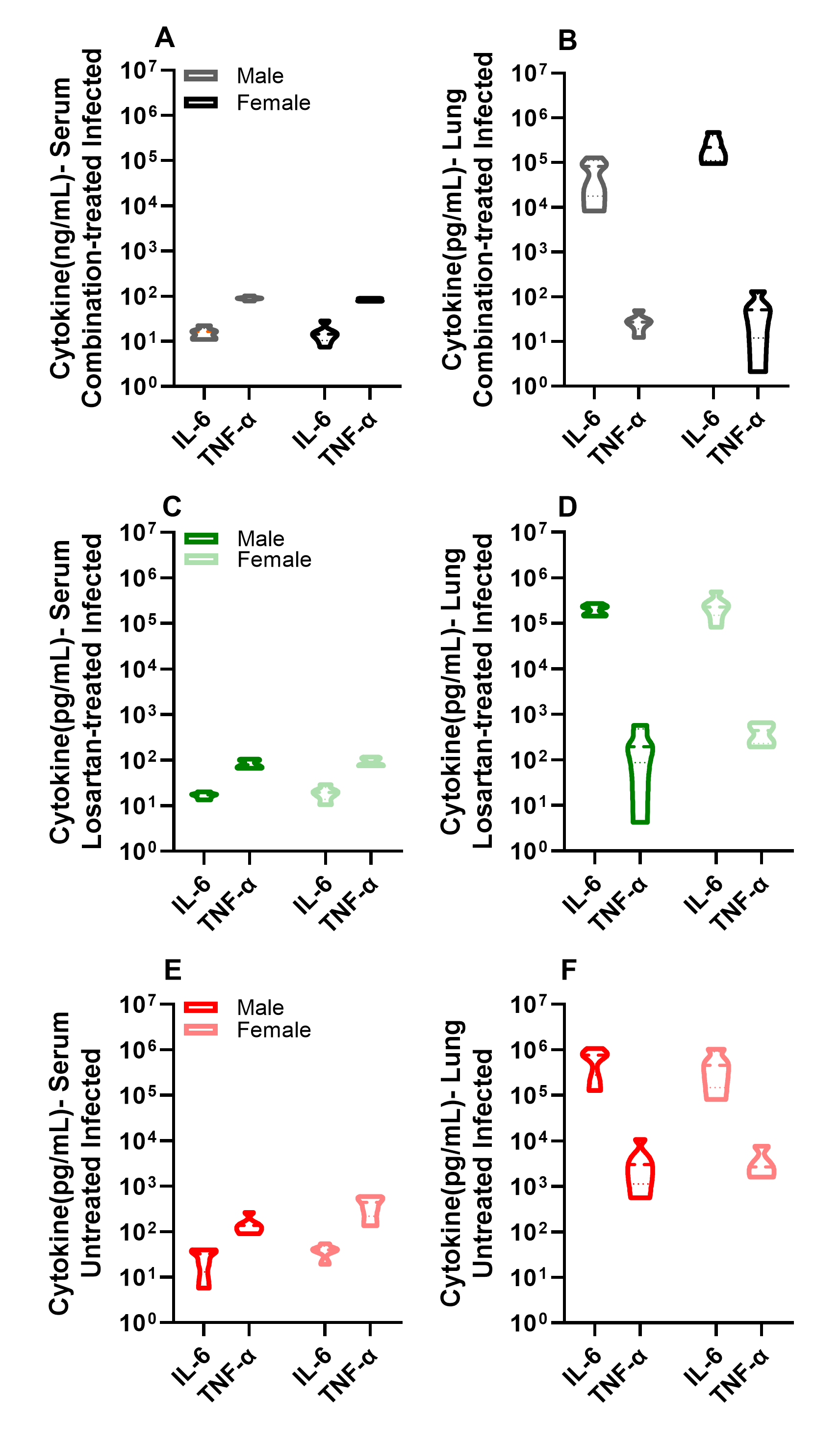

Supplement: Supplementary file 1 [file ijms-26-07663-s001.zip › SupFigureS4.tif]

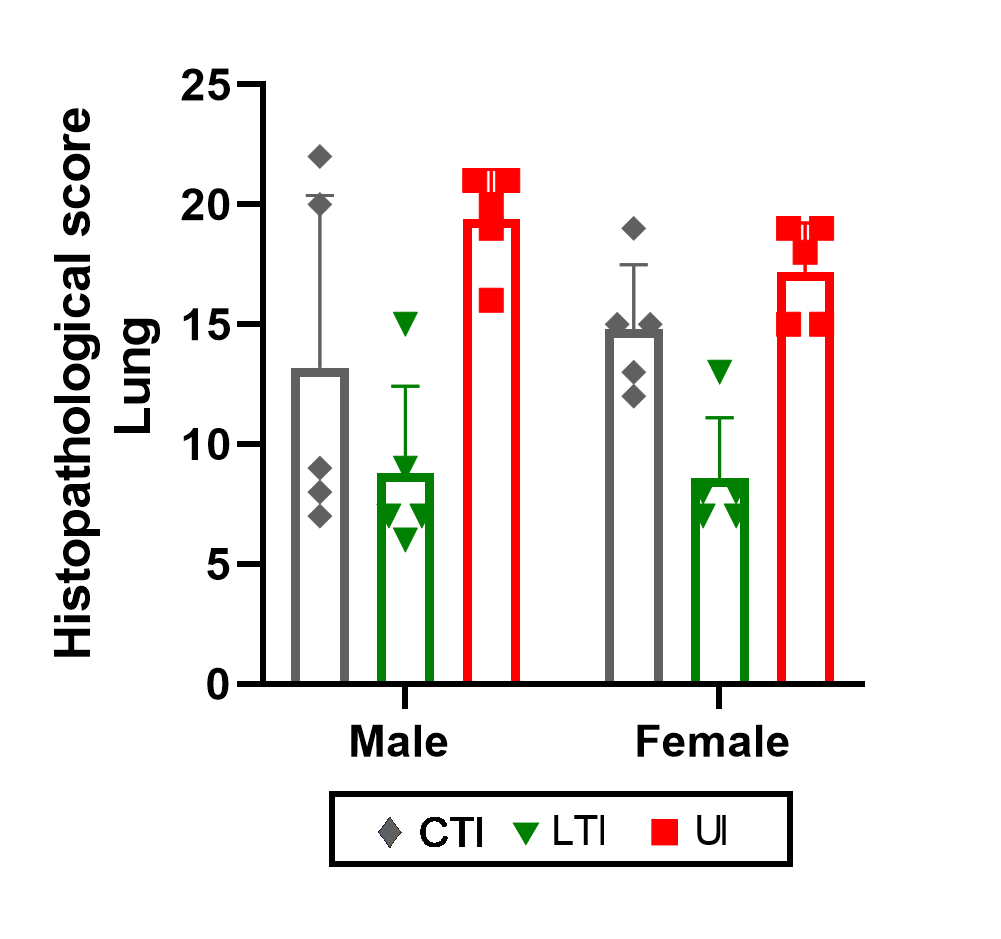

Supplement: Supplementary file 1 [file ijms-26-07663-s001.zip › SupFigureS5.tif]
